# Post-Stroke Outcomes of Patients with Chronic Obstructive Pulmonary Disease

**DOI:** 10.3390/brainsci12010106

**Published:** 2022-01-13

**Authors:** Aleksandra Szylińska, Katarzyna Kotfis, Marta Bott-Olejnik, Paweł Wańkowicz, Iwona Rotter

**Affiliations:** 1 Department of Medical Rehabilitation and Clinical Physiotherapy, Pomeranian Medical University, 71-204 Szczecin, Poland; pawelwankowicz@gmail.com (P.W.); iwrot@wp.pl (I.R.); 2 Department of Anesthesiology, Intensive Therapy and Acute Intoxications, Pomeranian Medical University, 71-204 Szczecin, Poland; katarzyna.kotfis@pum.edu.pl; 3 Neurology Department of a Regional Specialist Hospital in Gryfice, 72-300 Gryfice, Poland; omarta@poczta.onet.pl

**Keywords:** acute ischemic stroke, chronic obstructive pulmonary disease, COPD, delirium, complications, mortality, outcome

## Abstract

Introduction: Research has shown that patients with ischemic stroke and coexisting obstructive respiratory disorders have worse clinical status on admission and increased long-term mortality. Patients with chronic obstructive pulmonary disease (COPD) are at increased risk of stroke, and the risk is even greater after exacerbation of COPD. Moreover, COPD and stroke share major risk factors, which are advancing age and smoking. The aim of this study was to analyze the incidence of complications and mortality in acute ischemic stroke (AIS) patients with and without COPD. Material and methods: We analyzed prospectively collected data of 1022 patients with acute is-chemic stroke hospitalized in a district general hospital. The patients were divided into two groups—with coexisting COPD and without COPD. Results: Logistic regression analysis, which allowed for potential confounders, showed an association between coexisting COPD and the fol-lowing complications in acute ischemic stroke patients: heart failure (OR = 1.879, *p* = 0.048), atrial fibrillation (OR = 4.746, *p* = 0.012), delirium (OR = 2.803, *p* < 0.001), pneumonia (OR = 2.424, *p* = 0.005), bronchospasm (OR = 3.400, *p* = 0.023), and out-hospital mortality (OR = 2.656, *p* = 0.001). Conclusion: Patients presenting with acute ischemic stroke and coexisting COPD significantly more often had cardiac and pulmonary complications, as well as delirium following stroke. In a long-term follow-up, the probability of one-year survival was significantly lower in AIS patients with co-existing COPD.

## 1. Introduction

Chronic obstructive pulmonary disease (COPD) is a progressive respiratory disease characterized by persistently reduced airflow through the lungs with chronic inflammatory response, yet defined as a common, preventable, and treatable medical condition, with various degrees of severity and comorbidities [1]. It is the third most common disease contributing to death in the world [2,3]. Its pathophysiological hallmarks include chronic inflammation of the small airways that induces repeated damage and repair, which eventually result in the destruction of lung parenchyma, loss of elasticity, bronchiolar fibrosis and small airway obstruction, and emphysema with airspace dilatation [4]. Worsening deteriorating respiratory function contributes to increased dyspnea and patient deterioration [5]. 

COPD is commonly associated with different neurological and psychiatric comorbidities, including cerebrovascular and neurodegenerative disorders, as well as epilepsy, psychiatric and sleep disorders [6]. Stroke is the leading cause of disability in the world and the second leading cause of death [7]. About 30% of stroke survivors struggle with disability and require assistance in their basic daily activities for the rest of their lives, making stroke a major health, social, and economic problem [5,8]. Approximately 80–87% of all strokes are acute ischemic strokes (AIS), characterized by a sudden disruption of cerebral blood flow when it is blocked by thrombus, embolic thrombus or cerebral vascular insufficiency [5,8,9].

Stroke risk factors include diabetes mellitus, hypertension, atrial fibrillation, hypercholesterolemia, and lung disease; whereas smoking accounts for just over 60% of the risk of stroke, likewise in COPD. Thus, COPD and stroke share a major risk factor—smoking, which, unlike age, belongs to modifiable risk factors. In addition, studies have shown links between the occurrence of stroke and COPD—patients with COPD are at an increased risk of stroke, and moreover, this risk is greater after the exacerbation of COPD [5]. Stroke severity on admission and mortality among patients with COPD and bronchial asthma has been evaluated by Hausler et al., who have shown that ischemic stroke patients with coexisting obstructive respiratory disorders had a worse clinical status on admission and higher long-term mortality [10].

Therefore, the aim of this study was to compare the outcomes after acute ischemic stroke in patients with and without coexisting COPD, in terms of the incidence of complications, clinical status, and disability at discharge, along with short-term and long-term mortality.

## 2. Materials and Methods

### 2.1. Study Population

**Figure 1:**
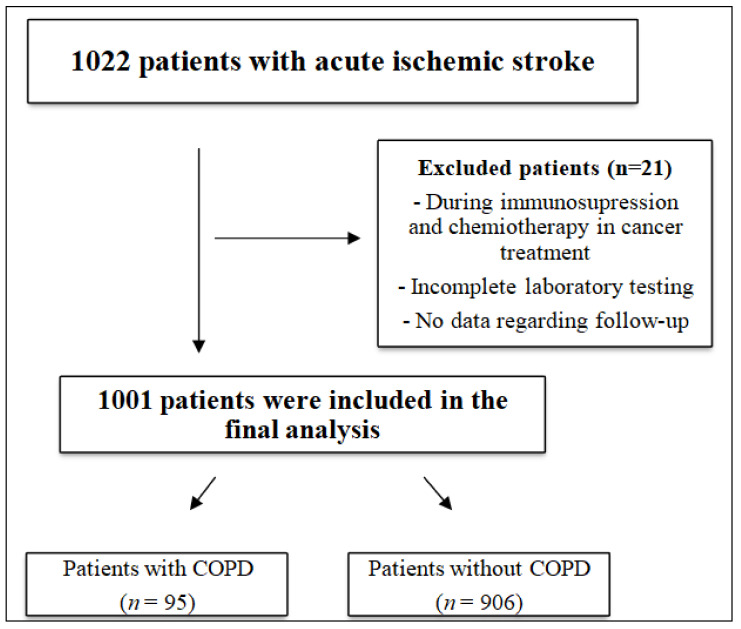
Study flowchart. Notes: COPD—chronic obstructive pulmonary disease; *n*—number of patients.

This study was an analysis of observational data from the Neurology Department of a district general hospital in Poland collected prospectively between June 2015 and March 2018. It included 1022 patients with acute ischemic stroke with symptom onset within 48–72 h prior to admission. Exclusion criteria included incomplete laboratory results (*n* = 6), no data regarding follow-up (*n* = 10), and coexisting hematological disorders (*n* = 5). A total of 21 patients were excluded from the study, and a total of 1001 patients were included in further analysis. Patients were divided into two groups, the first group included patients with ischemic stroke and coexisting COPD, and the second group included patients without coexisting COPD (Figure 1).

### 2.2. Data Collection

Data regarding demographics and comorbidities were obtained from the patient’s medical history and hospital electronic database system. Routine clinical and neurological evaluation of the patient was performed upon presentation to the hospital neurology department. On admission and at discharge, information was obtained regarding the severity of stroke according to the National Institute of Health Stroke Scale (NIHSS), and the degree of disability according to a modified Rankin Scale. In addition, Doppler ultrasonography of the carotid arteries and neuroimaging by computed tomography (CT) of the brain were performed. CT lesions were classified into one of four categories: no lesion, lesion < 2.5 cm, lesion > 2.5 cm (supratentorial and infratentorial), and lacunar lesion (supratentorial). Medical records provided information regarding the results of routine laboratory tests (blood count, C-reactive protein, troponin T, cholesterol, triglycerides, serum creatinine).

Follow-up data were extracted from medical records and by telephone follow-up after discharge. Hospital complications were divided into the following subgroups: cardiological complications (heart failure, myocardial infarction, sudden cardiac arrest, atrial fibrillation); renal complications (acute renal failure (ARF), exacerbation of chronic renal failure, hemofiltration in ARF, urinary tract infection (UTI)); neurological complications (seizures, delirium); pulmonary complications (respiratory failure, pneumonia, pulmonary embolism, bronchospasm) and other—bed sores and sepsis. Mortality was recorded as hospital mortality up to day 7 and telephone contact was used to obtain data on mortality at 30 and 90 days, and 1 year after stroke.

### 2.3. Design

The study was conducted according to the Strengthening the Reporting of Observational studies in Epidemiology (STROBE) guidelines for cohort studies (Supplementary Materials, Table S1) [11].

### 2.4. Ethical Issues

The study was performed in accordance with the Declaration of Helsinki. We obtained the approval of the Bioethical Committee of the Pomeranian Medical University: KBE-0012/84/03/19. The requirement to obtain informed consent was waived, as the study was performed as a routine clinical process of diagnosis and treatment performed in every patient admitted to the hospital. The data was analyzed anonymously.

### 2.5. Statistical Analysis

All statistical analyses were performed with the use of Statistica 13 software (StatSoft, Inc. Tulsa, OK, USA). The data are presented with descriptive statistics. All the continuous variable data are presented as medians and means ± standard deviations (±SD); categorical variables are presented as numbers and percentages. We analyzed the qualitative data using a Chi-square test or Chi-square test with Yates’s correction for the groups with COPD and without COPD. A Mann Whitney U-test was used to compare continuous variables. Logistic regression analysis was performed for COPD and the odds ratios (OR) calculated. Multivariable logistic regression was adjusted for gender, smoking, ischemic stroke, transient ischemic attack earlier than last 30 days, chronic renal failure, extracardiac arteriopathy, Rankin score at admission, hemoglobin, C-reactive protein, and triglycerides. Sensitivity analysis was conducted to assess the robustness of the association to unmeasured or uncontrolled confounding. E-values were calculated for the odds ratio (OR) point estimates from studies of occurrence of COPD and post-stroke outcome [12]. Kaplan–Meier analysis was used to calculate the probability of survival one year after the stroke both for coexisting COPD and without COPD. Differences were regarded as statistically significant at *p* < 0.05.

## 3. Results

Characteristics of the 1001 patients with acute ischemic stroke are shown in Table 1, with 95/1001 (9.49%) patients with COPD and 906/1001 (90.5%) without COPD. Significantly more patients with COPD were male (*p* = 0.004) and smokers (*p* < 0.001). Differences between the groups were also observed for the presence of comorbidities, as more COPD patients had transient ischemic attack within 30 days prior to admission due to AIS (*p* = 0.014), history of ischemic stroke in the past (*p* = 0.040), chronic renal failure (*p* = 0.009), extracardiac arteriopathy (*p* < 0.001).

In addition, neurological data were evaluated (Table 2), and a difference was observed between the groups in the Rankin Scale score (*p* = 0.040).

In terms of laboratory data, the differences were seen for hemoglobin (*p* = 0.028), C-reactive protein (*p* < 0.001), and triglycerides (*p* = 0.003) (Table 3).

In the study group, the number of post stroke complications, in-hospital and out-hospital mortality, and length of hospitalization, according to the presence of COPD are shown in Table 4. A higher number of complications was observed in patients treated for AIS with COPD.

Univariate and multivariate logistic regressions were performed to quantify the strength of the association between the complications (Table 5). After considering potential confounders among patients with acute ischemic stroke in our analysis, an association between coexisting COPD and the following complications was obtained: heart failure (OR = 1.879, *p* = 0.048), atrial fibrillation (OR = 4.746, *p* = 0.012), delirium (OR = 2.803, *p* < 0.001), pneumonia (OR = 2.424, *p* = 0.005), bronchospasm (OR = 3.400, *p* = 0.023), disability on the Rankin scale (OR = 1.161, *p* = 0.044), and out-hospital mortality (OR = 2.656, *p* = 0.001). In addition, we used the E-value sensitivity analysis to quantify the potential implications of unknown confounders. We found that the unmeasured confounders were unlikely to explain the effect on relationships between COPD and risk factors as shown in Table 5.

**Figure 2:**
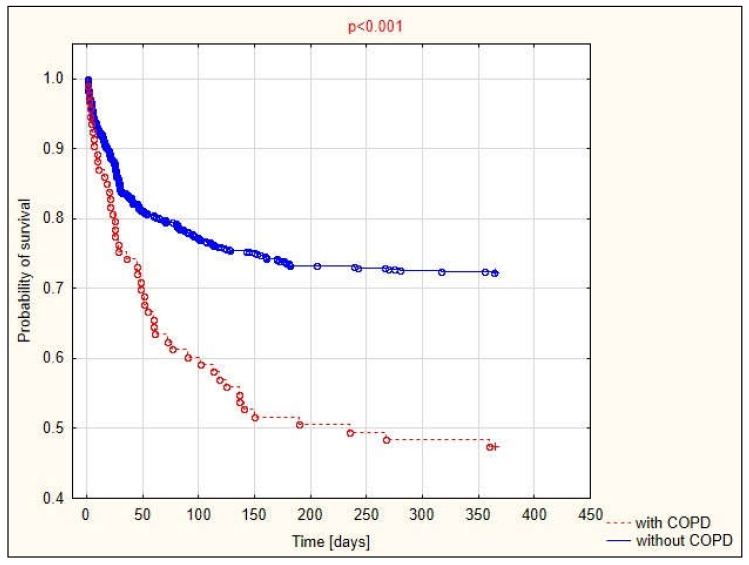
Annual probability of survival in stroke patients according to the presence of COPD.

Survival analysis according to the presence of COPD showed statistically significant differences between the survival times of acute ischemic stroke patients with coexisting COPD (Figure 2).

## 4. Discussion

Chronic obstructive pulmonary disease (COPD) and stroke are among the leading causes of death worldwide [13]. Despite literature data suggesting a frequent coexistence of these two conditions [14,15,16], there has not been any research on the actual prevalence of COPD among ischemic stroke patients. Our study showed that nearly 10% of patients with acute ischemic stroke had coexisting COPD. Similar results were obtained by Miguel (8.67%) [17].

It is well known that the most important risk factor of COPD, cigarette smoking, is also an established risk factor for ischemic stroke [18,19]. The results of our study also confirm this association. Thus, cigarette smoking appears to be a common link between COPD and ischemic stroke. Like other traditional stroke risk factors, cigarette smoking increases the propensity for ischemic stroke by impairing the ability of the cerebral circulation to meet the brain’s current oxygen demand. This occurs primarily because of structural changes in cerebral blood vessels that lead to their insufficiency and ultimately damage to the brain tissue through an interplay of many pathophysiological factors. These factors alter the structure of intracranial and extracranial blood vessels, promoting atherosclerosis, atrophy, and vessel stiffness [20]. Moreover, these structural abnormalities are usually accompanied by the impaired function of these vessels, which directly affects the regulation of cerebral blood flow. It is well documented that smoking causes endothelial dysfunction, which in turn is associated with an increased risk of stroke [21,22]. Smoking also impairs neurovascular coupling which is the primary adaptive mechanism that matches blood flow to neural activity. In addition to contributing to cerebrovascular disease, smoking modulates the risk of stroke by increasing the propensity of atherosclerotic plaque to rupture [23]. The prothrombotic effects of smoking are well documented [24]. Among other things, smoking increases platelet activation [25].

Although COPD is an independent risk factor for death in the general population, the association between COPD alone and the severity and mortality in ischemic stroke patients has not been widely addressed to date. Haeusler et al., conducted a study among patients with acute ischemic stroke according to the coexistence of COPD or bronchial asthma. The authors evaluated in-hospital and out-hospital mortality, and although they found no significant association between COPD and in-hospital mortality after ischemic stroke, they did find a correlation with out-hospital mortality [10]. Our analysis in patients with acute ischemic stroke and COPD has shown a strong association with long-term survival, but not with short-term mortality. 

Haeusler et al., assessed stroke severity with the use of NIHSS on admission and found no significant association in chronic inflammatory airway disease patients [10]. In our study, we likewise found no differences regarding the NIHSS at admission. In addition, we evaluated the degree of disability and independence in daily activities using the Rankin score. More disability was found in the group of COPD patients both at admission and discharge.

Our study provides additional evidence that ischemic stroke with coexisting COPD is a prognostically unfavorable combination than are stroke or COPD occurring alone. First, COPD causes systemic inflammation and oxidative stress, which are key mechanisms of stroke-related brain damage [5,26]. Thus, it can be predicted that COPD is also responsible for the severity of stroke outcomes by destroying other cells, tissues, and organs. Numerous disorders coexisting with COPD include cardiovascular diseases, neurological, metabolic, gastrointestinal, respiratory, or genitourinary disorders [27,28,29,30,31,32], where COPD leads to exacerbation of these conditions via a mechanism of systemic inflammatory response, and a vicious circle mechanism predisposes patients with these disorders to poorer long-term survival. 

Due to the symptoms of focal nerve tissue damage, such as bulbar and pseudobulbar syndromes, these patients are at risk of developing dysphagia, which leads to aspiration of food contents into the lungs resulting in the development of stroke-associated pneumonia (SAP). The incidence of SAP was higher among patients with COPD as has been show in our analysis. SAP is a common critical complication after ischemic stroke, increasing hospitalization time and cost, and is closely associated with high mortality. Risk factors for SAP include old age, male gender, dysphagia, severe ischemic stroke, impaired consciousness, speech impairment, pre-stroke disability, atrial fibrillation, heart failure, smoking, and COPD [33,34,35].

COPD may also increase the risk of stroke through hypoxemia. Patients with COPD may experience hypoxemia, and subsequent hyperventilation contributes to damage of the vascular wall. Hyperventilation may result in the development of metabolic abnormalities that predispose to cardiac arrhythmias. The study by Curkendall et al., demonstrated that abnormal lung function induced by COPD may result in paroxysmal atrial fibrillation, a condition predisposing to ischemic stroke [36].

## 5. Limitations

This study is not without limitations. It is a single-center observational series, so it would be useful to extend the research into a multicenter study. The study includes only patients with acute ischemic stroke, and so the conclusions cannot be generalized to all stroke patients. The differentiation of ischemic stroke for acute/subacute/chronic lesion was done based on a CT scan not magnetic resonance imaging (MRI) scan. In a future study, it should be considered to use images provided by an MRI scan. Further prospective studies with a larger sample size are needed.

## 6. Conclusions

Patients with ischemic stroke and coexisting COPD were significantly more likely to present a higher degree of disability at hospital discharge and a higher incidence of post-stroke delirium, as well as cardiac and pulmonary complications. In a long-term follow-up, the probability of one-year survival was significantly lower in stroke patients with COPD.

## Figures and Tables

**Table 1:** Demographic data and comorbidities in the stroke patients in relation to the presence of COPD.

Variables	No COPD (*n* = 906)	COPD (*n* = 95)	*p*
Demographic data			
Age [years], mean ± SD; Me	72.08 ± 12.32; 71.0	71.55 ± 10.92; 71.0	0.570
Gender [male]—n, (%)	460 (50.77%)	63 (66.32%)	0.004 *
BMI [kg/m^2^], mean ± SD; Me	26.89 ± 4.69; 26.0	25.99 ± 4.79; 25.8	0.129
Smoking, *n* (%)	348 (38.41%)	69 (72.63%)	<0.001 *
Co-morbidities			
Arterial hypertensio*n*—n, (%)	787 (86.87%)	82 (86.32%)	0.880
Ischemic heart diseases—n, (%)	230 (25.39%)	28 (29.47%)	0.386
Myocardial infarction, *n* (%)	94 (10.38%)	13 (13.68%)	0.321
Myocardial infarction over the past 90 days, *n* (%)	9 (0.99%)	0 (0.00%)	0.686
NYHA III and IV, *n* (%)	35 (4%)	7 (7%)	0.176
TIA over the last 30 days, *n* (%)	112 (12.36%)	17 (17.89%)	0.125
TIA earlier than last 30 days, *n* (%)	89 (9.82%)	18 (18.95%)	0.014 *
Ischemic stroke, *n* (%)	193 (21.30%)	29 (30.53%)	0.040 *
Hemorrhagic stroke, *n* (%)	23 (2.54%)	1 (1.05%)	0.584
Changes in CT, *n* (%)	286 (31.57%)	38 (40.00%)	0.095
Acute renal failure on admission, *n* (%)	8 (0.88%)	1 (1.05%)	0.686
Chronic renal failure, *n* (%)	121 (13.36%)	22 (23.16%)	0.009 *
Chronic dialysis, *n* (%)	2 (0.22%)	0 (0.00%)	0.454
Impaired insulin tolerance, *n* (%)	41 (4.53%)	7 (7.37%)	0.326
Diabetes on oral medications, *n* (%)	190 (20.97%)	15 (15.79%)	0.234
Diabetes on insulin, *n* (%)	116 (12.82%)	13 (13.68%)	0.811
Gout, *n* (%)	60 (6.62%)	5 (5.26%)	0.769
Extracardiac arteriopathy, *n* (%)	388 (42.83%)	66 (69.47%)	<0.001 *
Atrial fibrillation, *n* (%)	264 (29.17%)	29 (30.53%)	0.782
ICA stenosis, *n* (%)	75 (39.68%)	12 (42.86%)	0.749

Legend: *n*—number of patients; SD—standard deviation; Me—median; COPD—chronic obstructive pulmonary disease; BMI—body mass index; TIA—transient ischemic attack; NYHA—New York Heart Association; CT—computed tomography; ICA—internal carotid artery; *—statistical significance.

**Table 2:** Neurological data in stroke patients according to the presence of COPD.

Variables		No COPD (*n* = 906)	COPD (*n* = 95)	*p*
Rankin score at admission, mean ± SD; Me		3.08 ± 1.59; 3.0	3.45 ± 1.62; 4.0	0.040 *
NIHSS on admission, mean ± SD; Me		11.22 ± 7.93; 8.0	12.72 ± 8.21; 10.0	0.062
Hemianopia, *n* (%)		327 (36.09%)	42 (44.21%)	0.119
Dysphasia, *n* (%)		495 (54.64%)	52 (54.74%)	0.985
Brainstem stroke, *n* (%)		43 (4.75%)	4 (4.21%)	0.984
Secondary hemorrhage, *n* (%)		33 (3.64%)	1 (1.05%)	0.304
**Paresis, (%)**	Right sided	434 (53.19%)	39 (44.32%)	0.257
	Left sided	364 (44.61%)	46 (52.27%)	
	Bilateral	18 (2.21%)	3 (3.41%)	
**CT scan, (%)**	No lesion	59 (6.51%)	4 (4.21%)	0.368
	Lesion < 2.5 cm	374 (41.28%)	33 (34.74%)	
	Lesion > 2.5 cm	450 (49.67%)	56 (58.95%)	
	Lacunar stroke	23 (2.54%)	2 (2.11%)	
**Cerebral artery area, *n* (%)**	Anterior	176 (19.66%)	19 (20.00%)	0.981
	Middle	590 (65.92%)	63 (66.32%)	
	Posterior	129 (14.41%)	13 (13.68%)	
**Carotid artery stenosis, *n* (%)**	No stenosis	31 (3.53%)	1 (1.09%)	0.162
	Stenosis < 50%	534 (60.89%)	47 (51.09%)	
	Stenosis 50–70%	204 (23.26%)	28 (30.43%)	
	Stenosis > 70%	48 (5.47%)	8 (8.70%)	
	Occlusion	60 (6.84%)	8 (8.70%)	
**Stroke treatment, *n* (%)**	Conservative	735 (81.13%)	84 (88.42%)	0.079
	Thrombolysis	171 (18.87%)	11 (11.58%)	
CEA/CAS intervention after stroke	No intervention	861 (95.03%)	92 (96.84%)	0.718
	CEA	44 (4.86%)	3 (3.16%)	
	CAS	1 (0.11%)	0 (0.00%)	
Decompressive craniectomy	no	904 (99.89%)	95 (100.00%)	0.167
	yes	1 (0.11%)	0 (0.00%)	

Legend: *n*—number of patients; SD—standard deviation; Me—median; COPD—chronic obstructive pulmonary disease; NIHSS—National Institutes of Health Stroke Scale; CT—computed tomography; CEA—carotid endarterectomy; CAS—carotid artery stenting; *—statistical significance.

**Table 3:** Laboratory data in stroke patients according to the presence of COPD.

Laboratory Data	No COPD (*n* = 906)			COPD (*n* = 95)			*p*
	Mean	±SD	Me	Mean	±SD	Me	
Glycemia 0 (mg/dL)	143.43	61.55	125.0	139.08	53.27	122.0	0.520
Leucocyte count (× 10^9^/L)	9.74	3.94	9.0	10.44	4.25	9.5	0.199
Neutrophil count (× 10^9^/L)	6.92	4.57	6.0	7.59	4.13	6.7	0.191
Lymphocyte count (× 10^9^/L)	2.00	1.30	1.9	2.49	6.38	1.7	0.369
Platelet count (× 10^9^/L)	238.49	79.89	227.5	253.32	140.49	229.0	0.740
Hemoglobin	13.87	1.79	13.9	13.35	2.05	13.4	0.028 *
Creatinine	1.07	0.64	0.9	1.08	0.52	0.9	0.403
CRP	17.64	41.86	3.0	29.67	53.77	7.7	<0.001 *
Aspartate aminotransferase	27.45	55.76	20.0	23.95	15.43	21.0	0.749
Alanine aminotransferase	24.44	38.36	18.0	21.27	13.82	17.5	0.624
Cholesterol	193.93	54.14	189.0	185.04	51.84	184.0	0.304
Triglyceride	142.68	87.21	121.0	121.07	76.25	99.0	0.003 *
Troponin T	35.60	107.92	11.0	23.98	26.56	15.4	0.060

Legend: *n*—number of patients; SD—standard deviation; Me—median; COPD—chronic obstructive pulmonary disease; CRP—C-reactive protein; *—statistical significance.

**Table 4:** Evaluation of complications and mortality in patients after stroke according to the presence of COPD.

Complications		No COPD (*n* = 906)	COPD (*n* = 95)	*p*
**Cardiological complications**				
**Heart failure, *n* (%)**		119 (13.13%)	27 (28.42%)	<0.001 *
**Myocardial infarction, *n* (%)**		31 (3.42%)	3 (3.16%)	0.871
**Sudden cardiac arrest, *n* (%)**		88 (9.71%)	12 (12.63%)	0.469
**Atrial fibrillation, *n* (%)**		12 (1.32%)	6 (6.32%)	0.002 *
**Renal complications**				
**Acute renal failure, *n* (%)**		8 (0.88%)	4 (4.21%)	0.019 *
**Exacerbation of CRF, *n* (%)**		10 (1.10%)	5 (5.26%)	0.006 *
**Hemofiltration in ARF, *n* (%)**		1 (0.11%)	0 (0.00%)	0.167
**Urinary tract infection, *n* (%)**		69 (7.62%)	2 (2.11%)	0.075
**Neurological complications**				
**Seizures, *n* (%)**		15 (1.66%)	3 (3.16%)	0.521
**Delirium, *n* (%)**		125 (13.80%)	30 (31.58%)	<0.001 *
**Other complications, *n* (%)**		41 (4.53%)	5 (5.26%)	0.947
**Pulmonary complications**				
**Respiratory failure, *n* (%)**		91 (10.06%)	14 (14.74%)	0.157
**Pneumonia, *n* (%)**		185 (20.42%)	42 (44.21%)	<0.001 *
**Pulmonary embolism, *n* (%)**		2 (0.22%)	1 (1.05%)	0.671
**Bronchospasm, *n* (%)**		21 (2.32%)	7 (7.37%)	0.012 *
**Bed sores, *n* (%)**		30 (3.31%)	0 (0.00%)	0.137
**Sepsis, *n* (%)**		10 (1.10%)	2 (2.11%)	0.721
**Mortality**				
**Mortality up to day 7, *n* (%)**		59 (6.51%)	9 (9.47%)	0.380
**Mortality up to day 30, *n* (%)**		143 (15.78%)	24 (25.26%)	0.027 *
**Mortality up to day 90, *n* (%)**		204 (22.52%)	38 (40.00%)	<0.001 *
**Mortality up to 1 year, *n* (%)**		253 (27.92%)	51 (53.68%)	<0.001 *
**Hospitalization length (days); mean ± SD, Me**		10.62 ± 6.85; 9.0	11.84 ± 8.49; 10.0	0.015 *
**Scale at discharge**				
**NIHSS; mean ± SD, Me**		10.59 ± 12.83; 5.0	14.13 ± 13.63; 9.0	0.001 *
**Rankin score; mean ± SD, Me**		2.56 ± 2.23; 2.0	3.31 ± 2.15; 3.0	0.001 *
**Outcome**	In-hospital death, *n*, (%)	90 (9.93%)	14 (14.74%)	0.144
	Discharged home, *n*, (%)	541 (59.71%)	43 (45.26%)	0.007 *
	Nursing home, *n*, (%)	79 (8.72%)	13 (13.68%)	0.111
	Rehabilitation facility, *n*, (%)	180 (19.87%)	25 (26.32%)	0.138
	Another ward, *n*, (%)	16 (1.77%)	0 (0.00%)	0.381

Legend: *n*—number of patients; SD—standard deviation; Me—median; COPD—chronic obstructive pulmonary disease; ARF—acute renal failure; CRF—chronic renal failure; NIHSS—National Institutes of Health Stroke Scale; *—statistical significance.

**Table 5:** Multivariate logistic regression in patients after a stroke.

	COPD Not Adjusted					COPD Adjusted by **				
	*p*-Value	E-Value (OR)	OR	CI-95%	CI+95%	*p*-Value	E-Value (OR)	OR	CI-95%	CI+95%
**Scale at discharge**										
**NIHSS scale**	0.012 *	1.322	1.019	1.004	1.034	0.290	1.285	1.012	0.990	1.033
**Rankin score**	0.002 *	1.605	1.147	1.050	1.253	0.044 *	1.625	1.161	1.004	1.342
**Complications**										
**Heart failure**	<0.001 *	2.750	2.626	1.616	4.268	0.048 *	2.284	1.879	1.005	3.511
**Atrial fibrillation**	0.002 *	3.821	5.022	1.840	13.706	0.012 *	3.716	4.746	1.398	16.106
**Acute renal failure**	0.010 *	3.788	4.934	1.457	16.704	0.186	2.843	2.797	0.608	12.865
**Exacerbation of chronic renal failure**	0.004 *	3.805	4.978	1.665	14.882	0.642	1.968	1.479	0.284	7.692
**Delirium**	<0.001 *	2.889	2.884	1.799	4.623	<0.001 *	2.847	2.803	1.583	4.964
**Pneumonia**	<0.001 *	2.994	3.088	1.997	4.776	0.005 *	2.635	2.424	1.309	4.489
**Bronchospasm**	0.007 *	3.123	3.352	1.386	8.106	0.023 *	3.145	3.400	1.180	9.791
**Mortality up to day 30**	0.020 *	2.230	1.804	1.098	2.962	0.460	1.761	1.270	0.674	2.394
**Mortality up to day 90**	<0.001 *	2.557	2.294	1.479	3.559	0.134	2.053	1.577	0.869	2.861
**Mortality up to 1 year**	<0.001 *	2.945	2.992	1.949	4.593	0.001 *	2.767	2.656	1.456	4.847
**Hospitalization length**	0.113	1.323	1.019	0.995	1.044	0.988	1.000	1.000	0.971	1.030

Legend: *n*—number of patients; OR—odds ratio; CI—confidence interval; COPD—chronic obstructive pulmonary disease; *p*—the value of statistical significance; E-value—the potential index of unmeasured confounding; NIHSS—National Institutes of Health Stroke Scale; *—statistical significance. Notes: ** COPD adjusted by gender, smoking, ischemic stroke, TIA more than 30 days ago, chronic renal failure, extracardiac arteriopathy, ranking score at admission, hemoglobin, C-reactive protein, and triglyceride.

## Data Availability

All data that support the findings of this study are available upon request from the corresponding author.

## Supplementary Materials

The following are available online at https://www.mdpi.com/article/10.3390/brainsci12010106/s1, Table S1: STROBE Statement—checklist of observational studies.
